# Diagnosis of pulmonary nodules by DNA methylation analysis in bronchoalveolar lavage fluids

**DOI:** 10.1186/s13148-021-01163-w

**Published:** 2021-10-07

**Authors:** Lei Li, Zhujia Ye, Sai Yang, Hao Yang, Jing Jin, Yingying Zhu, Jinsheng Tao, Siyu Chen, Jiehan Xu, Yanying Liu, Weihe Liang, Bo Wang, Mengzhu Yang, Qiaoyun Huang, Zhiwei Chen, Weimin Li, Jian-Bing Fan, Dan Liu

**Affiliations:** 1 Department of Respiratory and Critical Care Medicine, West China Hospital, Sichuan University, No.37 Guoxue Alley, Wuhou District, Chengdu, 610041 Sichuan China; 2 AnchorDx. Medical Co., Ltd. Unit 502, 3rd Luoxuan Road, International Bio-Island, Guangzhou, 510300 Guangdong China; 3 AnchorDx, Inc., 46305 Landing Pkwy, Fremont, CA 94538 USA; 4 Department of Pathology, School of Basic Medical Science, Southern Medical University, 1838 ShaTai Road, Guangzhou, 510515 China

**Keywords:** Pulmonary nodules, Methylation markers, Diagnosis, Bronchoalveolar lavage fluid

## Abstract

**Background:**

Lung cancer is the leading cause of cancer-related mortality. The alteration of DNA methylation plays a major role in the development of lung cancer. Methylation biomarkers become a possible method for lung cancer diagnosis.

**Results:**

We identified eleven lung cancer-specific methylation markers (*CDO1, GSHR, HOXA11, HOXB4-1, HOXB4-2, HOXB4-3, HOXB4-4, LHX9, MIR196A1, PTGER4-1,* and *PTGER4-2*), which could differentiate benign and malignant pulmonary nodules. The methylation levels of these markers are significantly higher in malignant tissues. In bronchoalveolar lavage fluid (BALF) samples, the methylation signals maintain the same differential trend as in tissues. An optimal 5-marker model for pulmonary nodule diagnosis (malignant vs. benign) was developed from all possible combinations of the eleven markers. In the test set (57 tissue and 71 BALF samples), the area under curve (AUC) value achieves 0.93, and the overall sensitivity is 82% at the specificity of 91%. In an independent validation set (111 BALF samples), the AUC is 0.82 with a specificity of 82% and a sensitivity of 70%.

**Conclusions:**

This model can differentiate pulmonary adenocarcinoma and squamous carcinoma from benign diseases, especially for infection, inflammation, and tuberculosis. The model’s performance is not affected by gender, age, smoking history, or the solid components of nodules.

**Supplementary Information:**

The online version contains supplementary material available at 10.1186/s13148-021-01163-w.

## Introduction

Based on the published data in 2020 [1], lung cancer is one of the most dangerous malignant tumors for human health and life, with the highest mortality rates, and the 5-year relative survival rate for lung cancer is only 19%. However, if lung cancer can be diagnosed at the localized stage, especially for non-small cell lung cancer (NSCLC) on stage IA, the 5-year relative survival rate can achieve 92%. Therefore, it is an effective and essential way to prolong lung cancer patients’ lives by early diagnosis with appropriate treatments.

Currently, the clinical detection of lung cancer mainly adopts low-dose computed tomography (LDCT). The application of LDCT increases the detection rate of pulmonary nodules and reduces the mortality of lung cancer. However, it is hard to use LDCT alone for differentiating malignant nodules from benign. According to the data from the National Lung Screening Trial (NLST) test, the false positive rate of LDCT reached 96.4%, which can lead to an increase in unnecessary treatments [2].

The present standard clinical diagnosis methods of lung cancer include transbronchial lung biopsy, percutaneous aspiration biopsy of the lung, bronchoalveolar lavage fluid (BALF) [3, 4], and liquid biopsies (blood [5] or sputum [6]). Transbronchial lung biopsy and percutaneous aspiration biopsy of the lung are invasive diagnostic techniques and have limitations in sampling bronchoscopically invisible tumors. Nevertheless, bronchoalveolar lavage (BAL) can overcome these issues by sampling tumors by washing their surfaces [7]. Sampling BALFs is a routine operation during bronchoscopy in individuals with suspected lung cancer [8, 9]. BAL has advantages of large sampling volumes and multiple wash times in one operation, which improve the specificity and sensitivity for lung cancer detection [10]. It is a simple and less invasive diagnostic technique [11], which can be ideal for diagnosing pulmonary nodules in the high-risk lung cancer population. Although liquid biopsies, especially tests based on blood, become popular for early cancer screening and diagnosis due to their minimal invasiveness/non-invasiveness, high compliance, and simple operation. However, the lack of tissue specificity and low sensitivity are huge challenges for blood-based tests. The sputum test can diagnose squamous cell carcinoma since sputum is mainly coughed up from the central atmospheric channel but may not be suitable for detecting adenocarcinoma that often occurs in the lung periphery [12].

DNA methylation is an epigenetic modification that is important for human development and diseases [13]. Aberrant DNA methylation can be causally involved in cancer progression by multiple mechanisms, such as inactivating tumor-suppressor genes [14]. With the development of highly sensitive techniques for DNA methylation detection, the aberrant methylation status of CpG islands becomes an attractive biomarker for cancer diagnosis [15, 16].

In this study, we selected an optimal 5-marker model from all the possible combinations of eleven lung cancer-specific DNA methylation markers. The results from the test set of tissue and BALF samples indicated that the methylation signals in BALF samples are derived from pulmonary tissues. The model was further validated in an independent data set containing only BALF samples and had great potential for differentiating benign and malignant pulmonary nodules.

## Methods and materials

### Study design

**Fig. 1:**
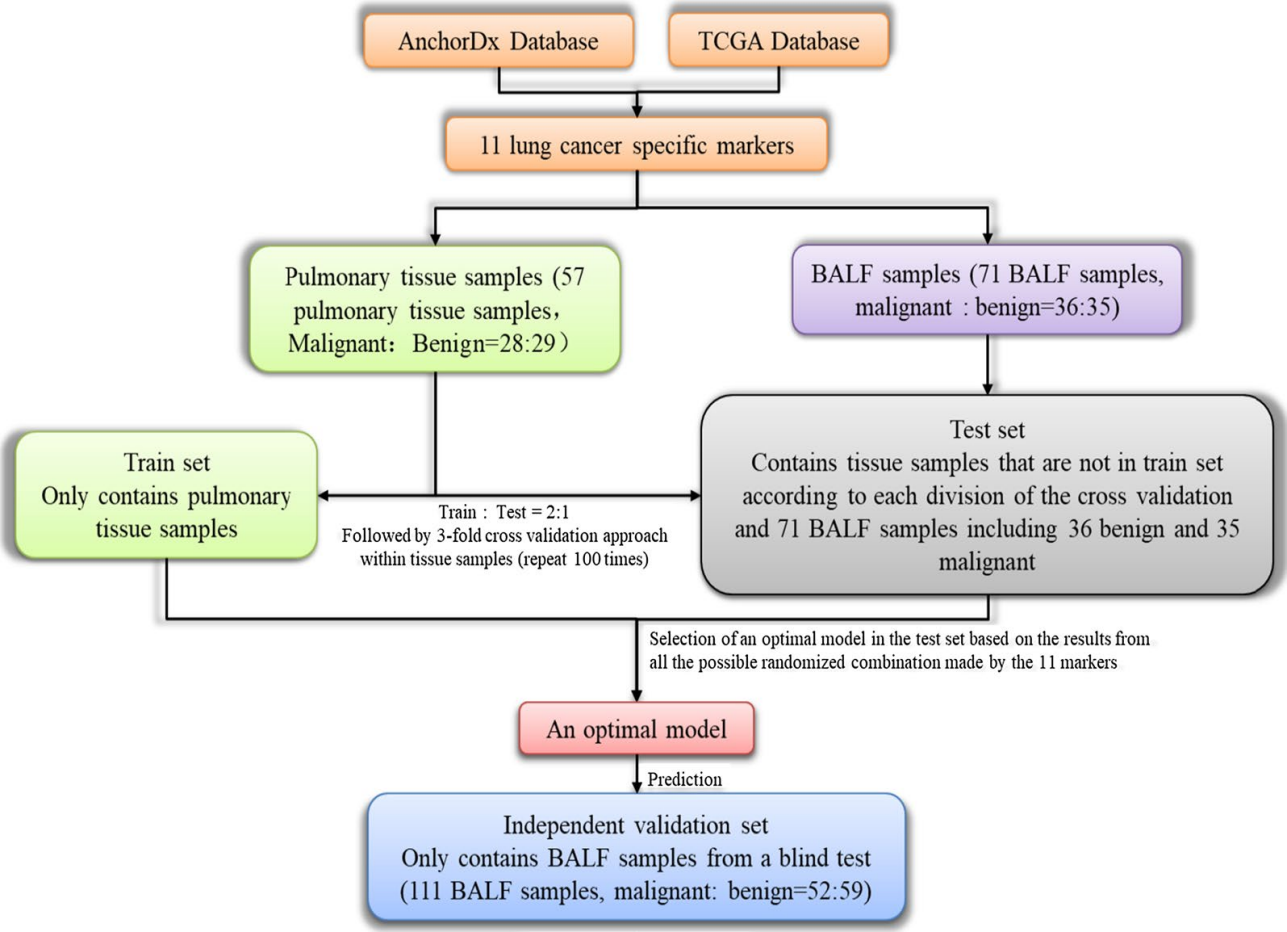
Study design

The study design is described in Fig. 1. Over 100 lung cancer-specific DNA methylation markers had been pre-selected from the in-house database [17] and public resources (e.g., TCGA Database). Considering the signal intensities (high sensitivity), noise level (specificity), and signal complementarities (low correlations), eleven markers (*CDO1, GSHR, HOXA11, HOXB4-1, HOXB4-2, HOXB4-3, HOXB4-4, LHX9, MIR196A1, PTGER4-1,* and *PTGER4-2*) were chosen for further investigations.

A total of 57 tissue (Malignant: Benign = 28:29) and 181 BALF samples (Malignant: Benign = 86:95) were enrolled to investigate these eleven candidate markers.

A three-segment train/test/independent validation set data division was applied to build and validate the model. In the first step, all the tissue and 71 BALF samples (Malignant: Benign = 35: 36) were used to construct the model. Tissue samples were divided by a threefold cross-validation (CV) approach, which was repeated 100 times. For each threefold CV division, two of the divisions were combined to form a train set, and the remaining one was used as a test set. Thus there were three possible combinations for each division, which created 300 different splits in this process. Each test set with pure tissue samples was then combined with the 71 BALF samples to form a complete test set. Considering the key performance indicators including but not limited to significances from the DMRs statistical tests, the area under curve (AUC) values along with overall and by stage sensitivity (Sn) under 80% specificity (Sp), an optimal model was chosen from all the possible combinations ($\sum_{i=1}^{n}{C}_{n}^{i}=\sum_{i=1}^{n}\frac{n!}{i!\left(\; n-i\right)!}\, , n\,=\,11$) of the eleven markers. In the second step, an independent data set of 110 BALF samples (Malignant: Benign = 51:59) was used to validate the selected optimal model.

### Available data from TCGA database

Methylation data (single-site beta value) from The Cancer Genome Atlas (TCGA) program with clinical information were downloaded from Genomic Data Commons (GDC) data portal (https://portal.gdc.cancer.gov/). The clinical features of the 816 patients used are listed in Additional file 1: Tables S1, which includes 446 lung adenocarcinoma (LUAD) patients (446 tumor and 23 normal tissues) and 370 lung squamous carcinoma (LUSC) patients (370 tumor and 40 normal tissues).

### Patients and samples collections

The specimens, formalin-fixed paraffin embedded (FFPE) pulmonary tissue and BALF samples, were collected at West China Hospital from 2015 to 2018. This study was approved by the ethics committee of West China Hospital, and written consent was collected from each participant.

The tissue samples were derived from 57 patients receiving lung tissue resection, including 28 cancer and 29 benign disease patients (Table 1). BALF samples were collected from 186 patients receiving fiberoptic bronchoscopy examinations. Among them, 90 were diagnosed with lung cancer following surgical biopsy, including 13 squamous cell carcinomas, 63 adenocarcinomas, one small cell lung cancer, and 13 unclassified lung cancer. The other 96 patients were confirmed as lung benign diseases, including pulmonary infection, inflammation, tuberculosis, and hamartoma, etc. Only 182 samples were used for further data analysis due to the quality control failures of four samples (Table 2). Within these BALF samples, 111 formed an independent sample set to validate the model's performance (Additional file 1: Table S2).

**Table 1:** Characteristics of patients receiving lung tissue resection

Clinical features	Lung cancer	Benign diseases	Total
*Age*			
> 60	12 (42.86%)	4 (13.79%)	16 (28.07%)
≤ 60	16 (57.14%)	25 (86.21%)	41 (71.93%)
*Sex*			
Male	14 (50%)	12 (41.38%)	26 (45.61%)
Female	14 (50%)	17 (58.62%)	31 (54.39%)
*Smoking history*			
Current	7 (25%)	6 (20.69%)	13 (22.81%)
Former	3 (10.71%)	1 (3.45%)	4 (7.02%)
Never	10 (35.71%)	19 (65.52%)	29 (50.88%)
Unknown	8 (28.57%)	3 (10.34%)	11 (19.30%)
*Tumor stage*			
Stage I	16 (57.14%)		
Stage II	3 (10.71%)		
Stage III	8 (28.57%)		
Stage IV	1 (3.57%)		
*Histology subtype*			
Adenosquamous carcinoma	25 (89.29%)		
Squamous cell carcinomas	3 (10.71%)		
Hamartoma		4 (13.79%)	
Infection		7 (24.14%)	
Inflammation		3 (10.34%)	
Tuberculosis		12 (41.38%)	
unknown		3 (10.34%)	
Total	28 (49.12%)	29 (50.88%)	57

**Table 2:** Characteristics of patients receiving fiberoptic bronchoscopy examination

Clinical features	Lung cancer	Benign diseases	Total
*Age*			
> 60	45 (50%)	26 (28.9%)	71 (38.17%)
≤ 60	45 (50%)	70 (77.8%)	115 (61.83%)
*Sex*			
Male	55 (61.1%)	56 (62.2%)	111 (59.68%)
Female	35 (38.9%)	40 (44.4%)	75 (40.32%)
*Smoking history*			
Current	23 (25.6%)	21 (23.3%)	44 (23.66%)
Former	25 (27.8%)	16 (17.8%)	41 (22.04%)
Never	42 (46.7%)	59 (65.6%)	101 (54.30%)
Unknown			
*Tumor stage*			
Stage I	54 (60.00%)		
Stage II	4 (4.44%)		
Stage III	7 (7.78%)		
Stage IV	13 (14.44%)		
unknown	12 (13.33%)		
*Histology subtype*			
Adenosquamous carcinoma	63 (70.0%)		
Small cell carcinoma	1 (1.1%)		
Squamous cell carcinomas	13 (14.4%)		
unknown	13 (14.4%)		
Atypical adenomatous hyperplasia		5 (5.2%)	
Hamartoma		6 (6.3%)	
Infection		13 (13.5%)	
Inflammation		49 (51%)	
Tuberculosis		14 (14.6%)	
Unknown		9 (9.4%)	
Total	90 (48.39%)	96 (51.61%)	186

Based on the smoking history, we divided the patients into two groups, “Non-smoking” and “Smoking”. “Non-smoking” was referred to the patients who have never smoked, while the “Smoking” group contained both current and former smokers.

According to nodule size, the largest diameter was determined by either LDCT or surgery.

For the analysis of solid components in nodules, the nodules were defined as ground-glass opacity solid (GGO) nodules as long as containing non-solid components; on the contrary, the solid nodules only have solid components.

### DNA Extraction and bisulfite treatment

Cell pellets were collected from 5 ml of BALF samples with centrifugation at 5000×*g* for 5 min and stored at − 80 °C until use. Genomic DNA (gDNA) was isolated from the pellets using DNeasy® Blood & Tissue Kit (QIAGEN, Catalog No. 69506, Hilden, Germany). Pulmonary tissue gDNA was isolated from FFPE tissue samples using QIAamp DNA FFPE Tissue Kit (Qiagen, Cat# 56,404, Hilden, Germany). Genomic DNA (gDNA) was quantified by the Qubit™ dsDNA HS Assay Kit (Thermo Fisher Scientific, Cat# Q32854, Eugene, Oregon, USA) and analyzed by the Agilent High Sensitivity DNA Kit (Cat# 5067–4626, CA, USA) on a 2100 Bioanalyzer Instrument (Agilent) for fragment size. gDNA was treated by the EZ DNA methylation-Direct™ kit (Zymo Research, Catalog No. D5021, Irvine, CA, USA) to convert unmethylated cytosines into uracils.

### DNA methylation analysis

The DNA Methylation analysis was used the MethyLight approach [18, 19]. The bisulfite-treated DNA was used as a template for the following multiplex PCR assay [20]. The multiplex PCR was implemented by Q5U® Hot Start High-Fidelity DNA Polymerase (New England BioLabs, Catalog, No. M0515, MA, USA) and an LC Detect Panel (AnchorDx, China, Catalog No. LCME-BAL-001) including markers *CDO1, GSHR, HOXA11, HOXB4-1, HOXB4-2, HOXB4-3, HOXB4-4, LHX9, MIR196A1, PTGER4-1,* and *PTGER4-2*, in a thermal cycler (Thermo Fisher, USA, Catalog No. 4484073) at 98 °C for 30 s, 5 cycles at 98 °C for 15 s, 58 °C for 15 s and 68 °C for 15 s, 13 cycles at 98 °C for 15 s, 63 °C for 15 s and 68 °C for 15 s, and 68 °C for 5 min. The amplified products were quantified by multiplex quantitative real-time PCR [19] using Luna® Universal Probe qPCR Master Mix (New England BioLabs, Catalog No. M3004E, MA, USA) on Applied Biosystems 7500 Real-Time PCR (Life Technologies Holdings Pte Ltd, Blk 33, Mariling Industrial Estate Rd 3, Singapore). The reaction was performed at 95 °C for 5 min, 40 cycles at 95 °C for 15 s and 62 °C for 30 s, with fluorescent signals collected at the annealing/extension step (62 °C for 30 s). The methylation-specific primers and probes were designed by Beacon Designer version 8.14 for detecting the methylation signals of target markers (Table 3). $\Delta$ CT values ($\Delta \mathrm{CT}={Marker}_{CT}-{Actin}_{CT}$) from the Quantitative PCR were used to represent the methylation levels of each marker. Those values would be given a “z-score” transformation before training and testing in the machine-learning model.

**Table 3:** Information of target markers

Gene name	Chromosome location (hg19)	Cpg sites on TCGA datacase
*IHX9*	chr1:197889098–197889188	cg09076431
*GSHR*	chr3:172166143–172166236	cg07852825, cg15987088
*CDO1*	chr5:115152460–115152575	cg08516516, cg11036833, cg23180938
*PTGER4-1*	chr5:40681603–40681717	
*PTGER4-2*	chr5:40681829–40681912	cg27071460
*HOXA11*	chr7:27225175–27225261	cg15760840
*HOXB4-1*	chr17:46655336–46655421	cg14458834, cg21546671
*HOXB4-2*	chr17:46655488–46655610	cg08089301, cg09194159, cg14345497
*HOXB4-3*	chr17:46655771–46655862	cg02422694, cg07015911, cg12806763, cg19081437, cg24114154, cg26327071
*HOXB4-4*	chr17:46655935–46656053	cg21460081
*MIR196A1*	chr17:46711296–46711411	cg01452847

### Statistics analysis

The methylation level of the markers of the TCGA data set was represented with beta value, whereas the methylation level of the markers of the tissue and BALF data set was represented with $\Delta$CT values from the Quantitative Real-time PCR experiment. Those values representing methylation statuses would be given a z-score transformation before training and testing in the machine-learning model.

Uniform Manifold Approximation and Projection (UMAP) was used to summarize large multi-dimensional datasets with a much smaller number of dimensions (ideally 2) while retain most of the useful information in the data (“umap” package from programming language of python).

To determine the differences between groups in a statistical way, Wilcoxon Rank Sum test (“Wilcox.test” function with “paired” parameter set to “FALSE” from programming language of R) with false discovery rate (FDR) adjustment for the *p* value (the threshold used here was 0.05) was performed.

Logistic regression model (“LogisticRegression” function in “sklearn” package from programming language of python) was constructed using methylation markers as input features and pathology classes as a label (digitalize the labels using 1 for malignant, 0 for benign/normal).

The performance of the model was evaluated with Area Under Curve (AUC) value along with overall and by stage sensitivity (Sn) under fixed specificity (Sp) or specificity (Sp) under fixed sensitivity (Sn).

Cancer Score was the output probability of the logistic regression model:

$$\widehat{Y}=\frac{{e}^{{\beta }_{0}+{\beta }_{1}{X}_{1}+{\beta }_{2}{X}_{2}+\dots +{\beta }_{i}{X}_{i}}}{1+{e}^{{\beta }_{0}+{\beta }_{1}{X}_{1}+{\beta }_{2}{X}_{2}+\dots +{\beta }_{i}{X}_{i}}}$$

Notice that the domain of $\widehat{Y}$ is between 0 and 1, $\widehat{Y}$ represents the estimated probability of being in one binary outcome category versus the other, and ${e}^{{\beta }_{0}+{\beta }_{1}{X}_{1}+{\beta }_{2}{X}_{2}+\dots +{\beta }_{i}{X}_{i}}$ represents the linear regression equation for independent variables expressed in the logit scale. As long as we set the labels of the samples from the cancer/malignant group as 1, and the benign/normal group as 0, for the training set of the model, after the model training process, we could get a predicted probability after passing each test sample to the model. This could be served as an indicator of whether this sample was at risk of being cancer/malignant: if it is closer to 1, it should more likely be cancer/malignant; if it is closer to 0, it should be less likely [21–23]. In this paper, this probability from logistic regression was named as “cancer score”.

Multivariate logistic regression used the pathological class of the subjects as the dependent variable and the covariates listed in Table 5 as the predictors. All predictors were entered simultaneously into the regression.

Unless otherwise specified, all statistical tests were two-sided.

## Results

### DNA methylation signals of pre-selected markers

In the eleven lung cancer-specific methylation markers (*CDO1, GSHR, HOXA11, HOXB4-1, HOXB4-2, HOXB4-3, HOXB4-4, LHX9, MIR196A1, PTGER4-1,* and *PTGER4-2*), 21 CpG sites (cg09076431, cg07852825, cg15987088, cg08516516, cg11036833, cg23180938, cg27071460, cg15760840, cg14458834, cg21546671, cg08089301, cg09194159, cg14345497, cg02422694, cg07015911, cg12806763, cg19081437, cg24114154, cg26327071, cg21460081, and cg01452847) were found in the TCGA database. Compared to the adjacent normal tissue samples, the median beta value of each CpG site is significantly higher (*p* value < 0.001) in the tumor tissues of both lung cancer adenocarcinoma (LUAD) and lung squamous carcinomas (LUSC) (Additional file 2: Figure S1A). According to our previous data from pulmonary tissues [17, 24], these CpG sites also have consistent performance between malignant and benign tissue samples (Additional file 2: Figure S1C and S1E). The methylation signals of the adjacent normal or benign tissues are clustered into a group away from malignant tissues, which inferred the differential methylation signals of these CpG sites between malignant and adjacent normal/benign tissues (Additional file 2: Figure S1B, S1D and S1F). In addition, clustering of LUAD and LUSC together suggested that these CpG sites have consistent methylation status between these two groups.

### Validation of target DNA methylation markers

**Fig. 2:**
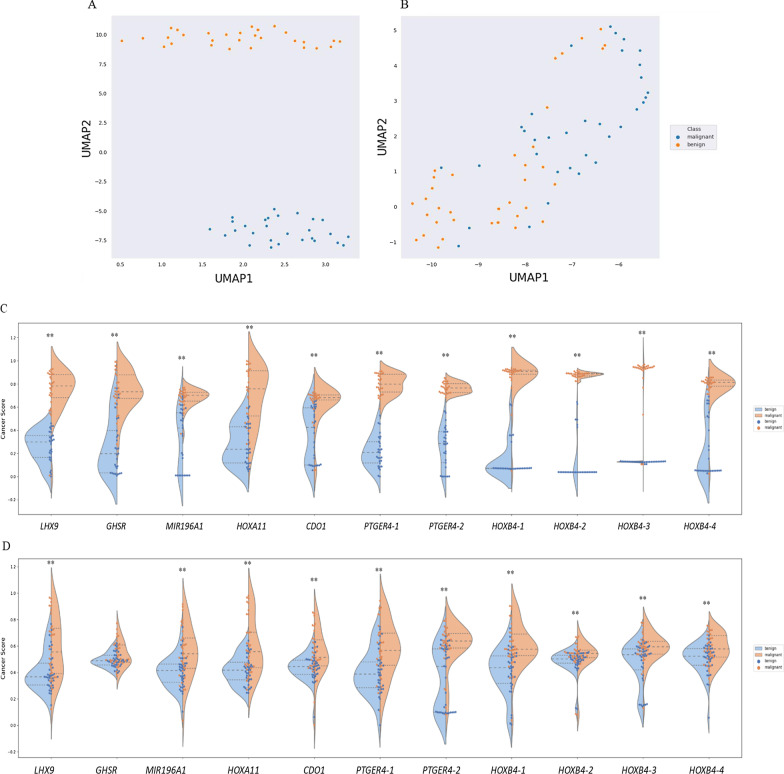
Methylation signals of 11 target markers in pulmonary tissues and bronchoalveolar lavage fluids (BALF). **a**, **b** The distribution of methylation signals of the 11 target markers in the pulmonary tissues and BALF, respectively. **c**, **d** The difference of the cancer score between benign and malignant nodules for the 11 candidate markers in the pulmonary tissues and BALF, respectively. Stars represent the difference of cancer scores between different stage cancers and benign diseases. “**”, *p* value < 0.01

Besides the performance on TCGA datasets, we further investigated the potentials of these CpG sites on the classification of malignant and benign pulmonary nodules using clinical pulmonary tissue and BALF samples, respectively. A concept, “caner score” (details described in Methods and Materials), was introduced to evaluate the capability of a marker or model for diagnosing pulmonary nodules. The cancer score of a malignant nodule should be closed to “1”, while the cancer score of a benign nodule should be closed to “0”. Therefore, the greater the difference of cancer scores in benign and malignant nodules is, the better the performance of a marker or model in differentiating benign and malignant nodules has. As shown in Fig. 2A, methylation signals of the malignant tissues are perfectly separated from the benign tissues. The cancer scores of the eleven markers are significantly different between benign and malignant tissues, especially for markers *LHX9, GHSR, PTGER4-1, PTGER4-2, HOXB4-1, HOXB4-2,* and *HOXB4-3* (Fig. 2C). Compared to the performance on tissues, the capability of the target markers on differentiating benign and malignant nodules is relatively weaker in BALF samples but still significant except for *GHSR* (*p* value: 0.069) (Fig. 2B). In BALF samples, the distributions of methylation signals of the target markers are scattered but still clustered into two groups (Fig. 2D). Followed by an approach of threefold cross-validation, the performance of individual markers was calculated (Table 4). The average area under curve (AUC) of *PTGER4-2, HOXB4-1, HOXB4-2, HOXB4-3, LHX9, MIR196A1, HOXA11,* and *CDO1* are all above 0.70. Among them, sensitivities (Sn) and specificities (Sp) of the markers, *PTGER4-2, HOXB4-1,* and *HOXB4-3* are both above 70%, which are 75% (Sn) / 73% (Sp), 74% (Sn) / 71% (Sp), and 71% (Sn) / 73% (Sp) at Youden’s index cutoffs, respectively. In summary, the lung cancer-specific methylation markers, *CDO1, GSHR, HOXA11, HOXB4-1, HOXB4-2, HOXB4-3, HOXB4-4, LHX9, MIR196A1, PTGER4-1,* and *PTGER4-2*, exhibit the potential of discriminating benign and malignant pulmonary nodules.

**Table 4:** Performance of individual marker in BALF

Gene	AUC	AUC.lower	AUC.upper	Sensitivity	Specificity
*PTGER4-1*	0.69	0.62	0.77	0.73	0.60
*PTGER4-2*	0.78	0.71	0.85	0.75	0.73
*HOXB4-1*	0.76	0.69	0.82	0.74	0.71
*HOXB4-2*	0.71	0.63	0.78	0.67	0.64
*HOXB4-3*	0.75	0.67	0.82	0.71	0.73
*HOXB4-4*	0.67	0.59	0.75	0.48	0.81
*IHX9*	0.71	0.64	0.79	0.59	0.77
*GSHR*	0.64	0.56	0.72	0.62	0.62
*MIR196A1*	0.71	0.63	0.78	0.58	0.78
*HOXA11*	0.70	0.62	0.78	0.55	0.79
*CDO1*	0.72	0.65	0.80	0.55	0.84

### Performance of the model on diagnosing pulmonary nodules

We further constructed model with all combinations of target markers to enhance performance compared to individual markers. The training data set, which only contained tissue samples, confirmed that the methylation signals are derived from pulmonary tissues. The test data set included both tissue and BALF samples to verify the signal consistency between tissue and BALF samples, adjust the model with appropriate parameters, and select the optimal model for clinical applications.

**Fig. 3:**
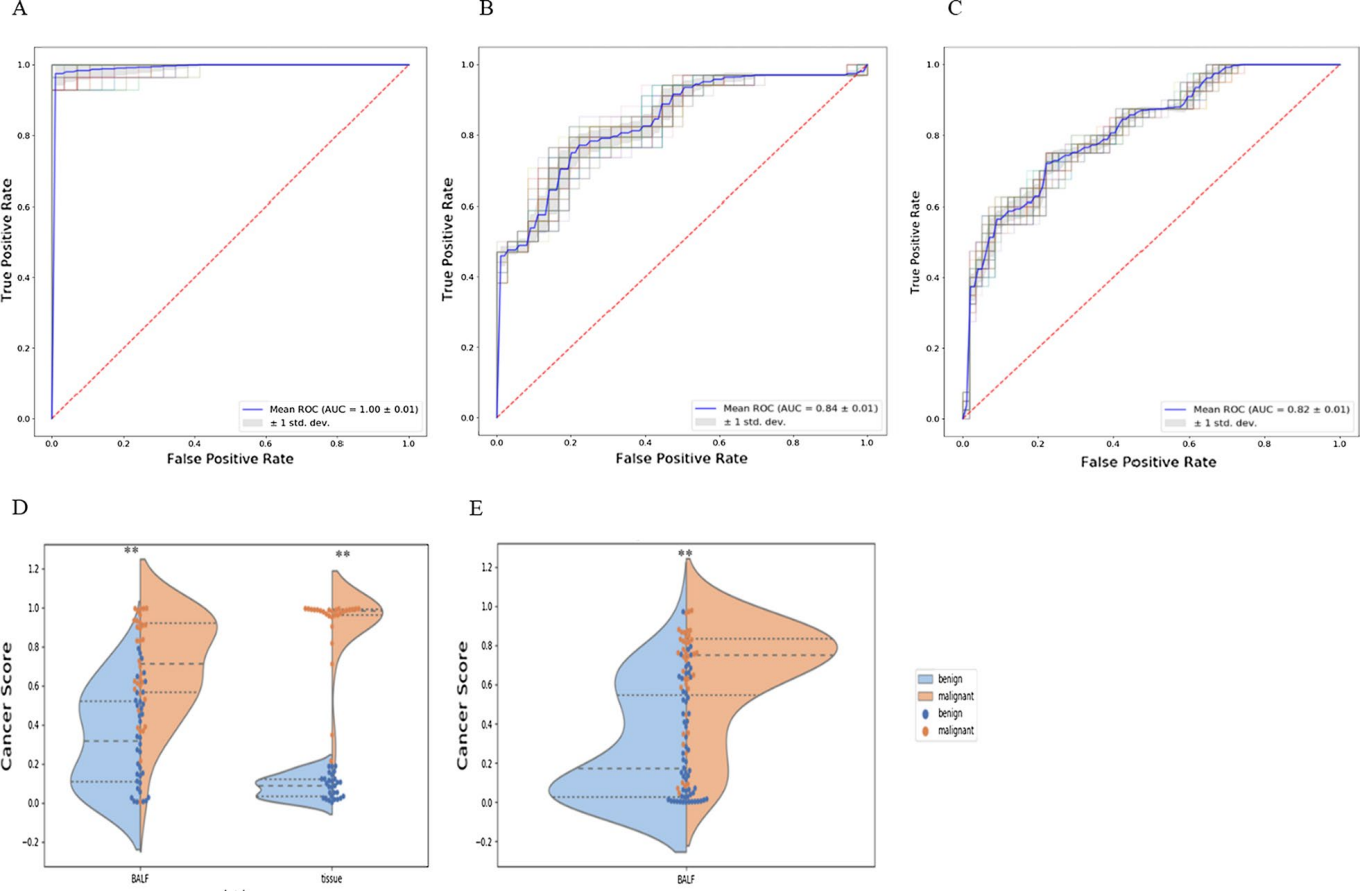
Performance of the optimal model on diagnosing benign and malignant pulmonary nodules. **a**, **b** The figures are the ROC curves of the model in the tissue samples and BALF samples, respectively, in the test set. **a** The figure is the ROC curve of the model in an independent validation set that only contains BALF samples. **d** The violin plots demonstrate the difference of cancer score between benign and malignant samples in the test set that contains pulmonary tissues and BALF samples. **e** The violin plots demonstrate the difference of cancer score between benign and malignant samples in an independent validation set that only contains BALF samples. Stars represent the difference of cancer score between different cancer stages tumors and benign diseases; “**”, *p* value < 0.01

**Fig. 4:**
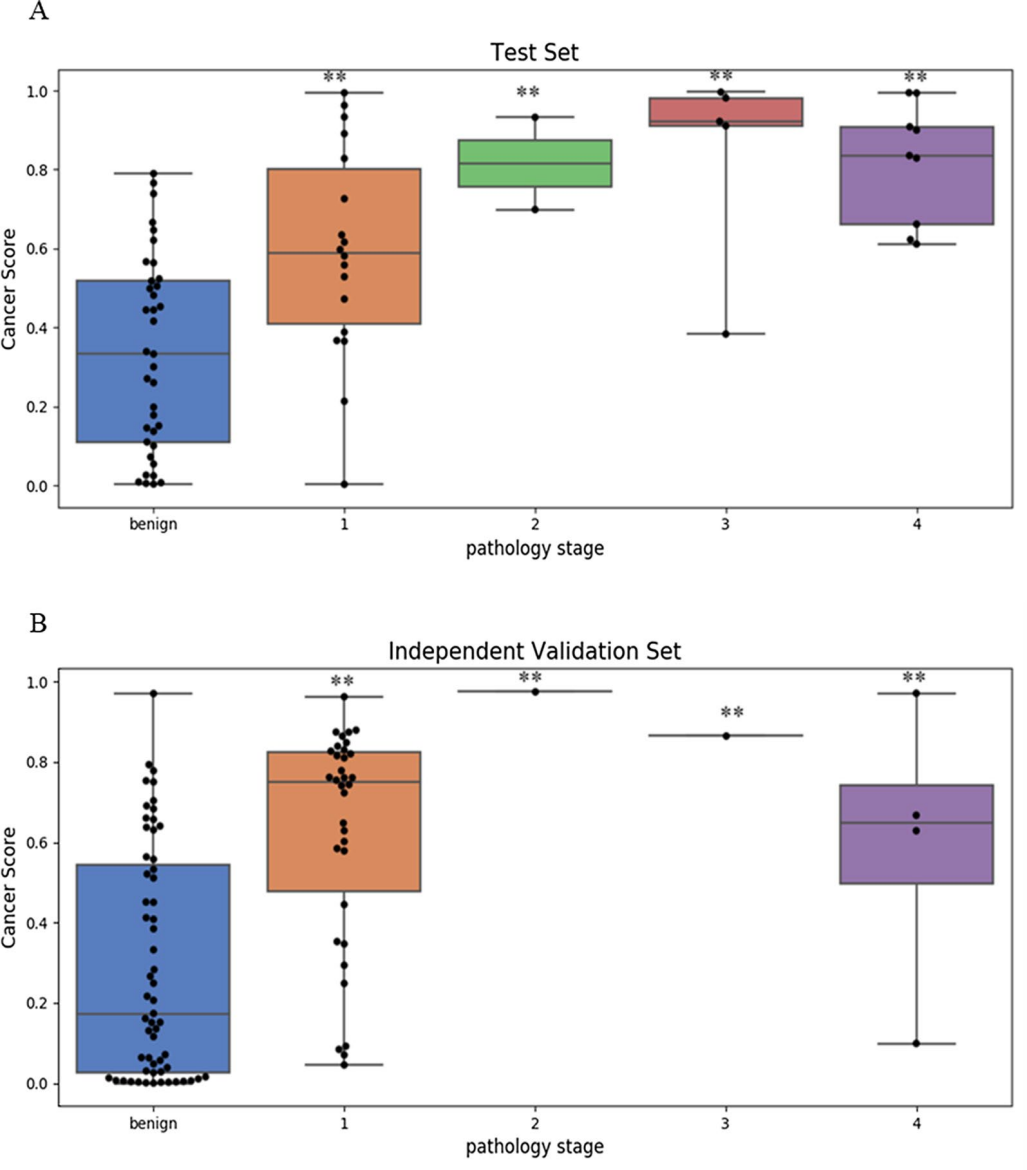
Performance of the optimal model in BALF on different pathology stage. The boxplots of **a**, **b** demonstrate the performance of the model in BALF on different pathology stage in the test set and the validation set, respectively. Stars represent the difference of cancer score between different cancer stages tumors and benign diseases; “**”, *p* value < 0.01

In the test data set, according to the results from all the possible combinations of the eleven markers, an optimal model (including markers *LHX9, GHSR, HOXA11, PTGER4-2,* and *HOXB4-3*) was selected for further analysis. In this model, the difference in cancer scores is significant between benign and malignant samples in both pulmonary tissues and BALF samples, which have less difference compared to tissue samples (Fig. 3d). Methylation levels of the target markers showed the same trend between tissue and BALF samples, which implied the methylation signals in BALF samples might be derived from pulmonary tissues. The AUC of the optimal model achieved 1.00 and 0.84 in tissue and BALF samples, respectively (Fig. 3a, b). The overall sensitivity is 82% at a specificity of 91% (Additional file 1: Table S3). The sensitivities mostly increase according to the stage status. The detection rates of Stage I lung cancer are 71.2% (Fig. 4 and Additional file 1: Table S4).

An independent validation set containing 111 BALF samples was set aside to evaluate the model’s diagnostic performance. In this independent sample set, the AUC is 0.82 (Fig. 3c and Additional file 1: Table S3), and the differences in cancer scores between benign and malignant samples are still significant (Fig. 3e). Under Youden’s index cutoff, the overall sensitivity can achieve 70% at a specificity of 82% (Additional file 1: Table S3). Moreover, the detection rate of Stage I lung cancer is 68.5% (Fig. 4 and Additional file 1: Table S4). In both test and validation data sets, this model showed great potential for differentiating pulmonary malignant and benign nodules.

### Performance of the model on pathological subtypes

To evaluate the model performance on different pathological subtypes, we analyzed pulmonary tumors (adenocarcinoma and squamous carcinoma) and benign diseases (pulmonary hamartoma, infection, inflammation, and tuberculosis).

For pulmonary tumors, this model can differentiate both adenocarcinoma and squamous carcinoma from benign diseases distinctly (The *p* values of difference in cancer scores either between benign nodules and adenocarcinoma or between benign nodules and squamous carcinoma are less than 0.01) (Additional file 2: Figure S2). Compared to adenocarcinoma, the cancer scores of squamous carcinoma are much closer to “1”, which indicated that this model has better performance on differentiating squamous carcinoma from benign diseases.

For benign diseases, the abilities to differentiate pulmonary hamartoma, inflammation, infection, or tuberculosis from tumors have been tested for the selected model (Additional file 2: Figure S2). Except for pulmonary hamartoma, differences between other benign diseases and malignant nodules are significant (*p* values < 0.01). Among these benign diseases, the cancer scores of infection and tuberculosis are not only remarkably below tumors, but also beneath other benign diseases, especially tuberculosis, whose cancer scores are closed to “0” (The *p* value of difference in cancer scores between tuberculosis and other benign diseases is less than 0.01). Therefore, the selected model is more capable of distinguishing malignant tumors from pulmonary infection and tuberculosis.

### Effect of physiological characteristics on diagnosing pulmonary nodules

Although the samples might differ in the age, gender, smoking history, or solid components of nodules, respectively, the cancer scores of benign nodules are consistently lower than the malignant nodules in this 5-marker model (Additional file 2: Figure S3A, S3B, S3C, S3E). The diagnostic power of this model was not affected by the factors mentioned previously, while it is better for the patients with larger target nodules size (Additional file 2: Figure S3D).

In the univariable analyses, the 5-marker model, age, and smoking history were as significant predictors for detecting pulmonary malignant nodule. In contrast, the multivariate analysis indicates only the 5-marker model as significant independent predictors of pulmonary malignant nodule detection (Table 5).

**Table 5:** Predictors of diagnosing pulmonary nodules

Analyzed variables	Univariable analysis		Multivariable analysis	
	OR (95% CI)	*p* value	OR (95% CI)	*p* value
Model: *LHX9* + *GHSR* + *HOXA11* + *PTGER4-2* + *HOXB4-3*	93.93 (27.178–382.05)	1.25E−11	43.15 (11.16–196.05)	2.17E−07
Male gender	1.22 (0.67–2.23)	0.5109	0.68 (0.23–2.00)	0.4927
Age	1.05 (1.02–1.08)	0.0007	1.03 (1.00–1.07)	0.0923
Positive smoking history	2.01 (1.11–3.65)	0.0212	1.70 (0.58–5.15)	0.3346
Ground glass opacity nodule type	1.34 (0.68–2.66)	0.3959	1.63 (0.68–4.07)	0.2850
Nodule size	1.05 (0.87–1.264)	0.6372	1.11 (0.88–1.43)	0.3851

## Discussion

To date, low-dose computed tomography (LDCT) is the primary strategy for the substantial reduction of lung cancer-related mortality in the long term within high-risk asymptomatic populations [25]. Two large randomized controlled trials, the US National Lung Screening Trial (NLST) [2] and the Dutch-Belgian lung-cancer screening trial (NELSON) [26], have proven that LDCT-based screening could statistically significantly reduce lung cancer-related mortality by more than 20% in high-risk individuals. While the high sensitivity of LDCT also brings a significant challenge in differentiating benign nodules from malignancy, which leads to a relatively high false-positive rate [27]. Suspected nodules detected by LDCT can be further diagnosed through lung biopsies, including bronchoscopy and percutaneous puncture. However, pulmonary peripheral and bronchoscopic invisible lesions are always challenges for lung biopsy diagnosis. The application of BALF is more likely to obtain tissue/cells released from peripheral or bronchoscopic invisible lesions, which would potentially solve these issues [28, 29].

Eleven lung cancer methylation-specific markers (*CDO1, GSHR, HOXA11, HOXB4-1, HOXB4-2, HOXB4-3, HOXB4-4, LHX9, MIR196A1, PTGER4-1,* and *PTGER4-2*) were selected from the previous studies [17, 24] based on their capabilities of differentiating pulmonary benign and malignant nodules on the tissue level. Twenty-one CpG sites in these eleven genes were further validated to have significantly different methylation levels between lung cancer and normal tissues in the TCGA database (Additional file 2: Figure S1A). *CDO1*, a tumor suppressor gene, plays a role in the oxidative stress response of cancer cells [30, 31]. *PTGER4* proteins belong to the G-protein coupled receptor family. As the hypermethylated markers, *CDO1* and *PTGER4* were detected using plasma and sputum samples in early-stage lung cancers [5, 32]. MiR196 gene family could play an essential role in regulating HOX gene expression, and their dysregulated expression in multiple cancers may function as both oncogenes and tumor suppressors [33, 34]. *MIR196A1* and *HOXA11* have been reported to be highly methylated in the bronchial washings of lung cancer patients*.* The methylation levels of *MIR196A1* were inversely associated with the duration of smoking cessation in healthy people [35]. *LHX9* encodes a transcription factor that might involve the control of cell differentiation of several neural cell types [36], but its methylation status in lung cancer has not been reported. Glutathione reductase (GSHR) is a biologically important enzyme involved in the protection against ROS [37, 38]. HOXB4 plays a vital role in proliferation, metastasis, and angiogenesis in cancers [39–43]. Moreover, *GSHR* and *HOXB4* were reported with differential methylations in lung adenocarcinoma [44].

To verify whether the methylation signals of these markers were derived from pulmonary tissues, we trained the model using pulmonary tissue samples only and then tested them on both pulmonary tissues and BALF samples. The trends of differential methylation signals between benign and malignant samples are consistent in both pulmonary tissues and BALF. Although BALF methylation signals can be traced back to pulmonary tissues, the less difference of cancer scores between benign and malignant samples (Fig. 2) might be owing to the less tumor burden or cancer cells in BALF samples compared to pulmonary tissues. Therefore, using both pulmonary tissue and BALF samples as a test set can preliminarily verify the model performance and adjust the model with appropriate parameters for the BALF application. Through further validation in an independent sample set, the selected model maintained robust performance for differentiating benign pulmonary nodules from malignancy. In particular, the sensitivity of stage I lung cancer was up to 71.2% and 68.5% in the test and independent validation set, respectively, which was of great clinical significance for diagnosing early-stage lung cancers.

In terms of two main subtypes of lung cancer, LUAD and LUSC, the selected model could effectively differentiate both of them, with a better diagnostic power for LUSC (Additional file 2: Figure S2A and S2B). Since LUSC is mainly associated with the central air tract while LUAD mainly occurs in the lung periphery, the different signal intensities may be ascribed to their locations. Even though it has a high chance to obtain cells from peripheral or subsegmental bronchioles where targeted tissues might locate through the current bronchoalveolar lavage procedure, the overall targeted cell amount is still limited. As artificial intelligence and robots for interventional surgery are continuously being developed, the first robot-assisted bronchoalveolar lavage procedure for bronchus surgeries has been reported [38]. The surgical robot can help the bronchoalveolar lavage procedure and reach smaller areas such as bronchial trees. This new approach may improve targeted tissue sampling and increase the test signals in the future.

This model has shown significant differences between pulmonary malignancy and benign diseases such as tuberculosis, infection, and inflammation. Among them, the diagnostic power of the model for tuberculosis and infection is better than other benign diseases. In our data, the methylation levels of hamartoma are higher than other benign diseases but are still lower than lung cancer tumors, yet not significantly. In the clinical diagnosis, certain pathology subtypes like pulmonary hamartoma could potentially utilize BALF inspection, lung cancer-specific marker tests of BALF samples, and AI-aided diagnosis by CT/LDCT scans to further improve the diagnostic accuracy [45].

Smoking has been reported to alter lung function, gene expression, and DNA methylation [46–49]. The accuracy of determining ground-glass opacity (GGO) lesions is relatively low using the traditional diagnostic techniques [50], and often differs depended on GGO components. Aging would change the statues of DNA methylation [51]. The differences of DNA methylation might contribute to the sex-related differences in cancer [52]. The selected model has strong performance on diagnosing pulmonary benign nodules from malignant nodules regardless of smoking status, GGO components, genders, and age.

## Conclusions

In summary, we have developed a DNA methylation test using BALF samples for diagnosing pulmonary nodules, especially on differentiating pulmonary infection, inflammation, and tuberculosis from malignancy. The model’s performance is not affected by gender, age, smoking history, and nodule component. It showed great potential for pulmonary nodule management in the clinical setting.

## Supplementary Information

**Additional file 1**. **Table S1**: Characteristics of the patient population from TCGA database. **Table S2**: Characteristics of the patients in the independent test set. **Table S3**: Performance of the optimal model in the test set and the independent validation set. **Table S4**: Performance on different cancer stages in the test set and the independent test set.

**Additional file 2**. **Figure S1**: Methylation signals of the target CpG sites in the TCGA and the in-house databases. **Figure S2**: Performance of the optimal model in BALF on different pathology subtypes. **Figure S3**: Effect of physiological characteristics on diagnosing pulmonary nodules.

## Data Availability

The datasets used and/or analyzed during the current study are available from the corresponding author on reasonable request.

## Abbreviations

BALF: Bronchoalveolar lavage fluid
AUC: Area under curve
LDCT: Low-dose computed tomography
Sn: Sensitivity
Sp: Specificity
TCGA: The Cancer Genome Atlas
FFPE: Formalin-fixed paraffin embedded
UMAP: Uniform manifold approximation and projection
LUAD: Lung cancer adenocarcinoma
LUSC: Lung squamous carcinomas
GGO: Ground-glass opacity
