# Supplementary material for: Diagnosis of pulmonary nodules by DNA methylation analysis in bronchoalveolar lavage fluids
Source: Clin Epigenetics. 2021 Oct 7;13:185. doi: 10.1186/s13148-021-01163-w (PMC8499516; doi:10.1186/s13148-021-01163-w)
Supplement: Supplementary file 1 — Additional file 1. Table S1: Characteristics of the patient population from TCGA database. Table S2: Characteristics of the patients in the independent test set. Table S3: Performance of the optimal model in the test set and the independent validation set. Table S4: Performance on different cancer stages in the test set and the independent test set. [file 13148_2021_1163_MOESM1_ESM.docx]

**Supplement Table 1. Characteristics of the patient population from TCGA database**

| **Clinical Features** | **TCGA-LUAD^*^** | **TCGA-LUSC^*^** | **All** |
| --- | --- | --- | --- |
| primary_solid_tumor | 446 | 370 | 816 |
| solid_tissue_normal | 23 | 40 | 63 |
| All | 469 | 410 | 879 |
| **Age （Year)** |  |  |  |
| ≤60 | 141 | 78 | 219 |
| >60 | 286 | 283 | 569 |
| unknown | 19 | 9 | 28 |
| **Sex** |  |  |  |
| female | 237 | 96 | 333 |
| male | 209 | 274 | 483 |
| **Race** |  |  |  |
| asian | 6 | 7 | 13 |
| black or african american | 48 | 24 | 72 |
| not reported | 49 | 65 | 114 |
| white | 343 | 274 | 617 |
| **Stage** |  |  |  |
| I | 238 | 172 | 410 |
| II | 110 | 135 | 245 |
| III | 73 | 56 | 129 |
| IV | 20 | 4 | 24 |
| unknown | 5 | 3 | 8 |
| **Survival Status** |  |  |  |
| Alive | 284 | 210 | 494 |
| Dead | 162 | 160 | 322 |
| **All** | **446** | **370** | **816** |

LUSC, lung squamous carcinoma;

LUAD, lung adenocarcinoma;

**Supplement Table 2. Characteristics of the patients in the independent test set**

| **Clinical Features** | **Benign** | **Malignant** | **Total** |
| --- | --- | --- | --- |
| **Age** |  |  |  |
| ≤60 | 18 (30.51%) | 28 (53.85%) | 46 (41.44%) |
| >60 | 41 (69.49%) | 24 (46.15%) | 65 (58.56%) |
| **Sex** |  |  |  |
| female | 26 (44.07%) | 19 (36.54%) | 45 (40.54%) |
| male | 33 (55.93%) | 33 (63.46%) | 66 (59.46%) |
| **Smoking History** |  |  |  |
| Current | 13 (22.03%) | 12 (23.08%) | 25 (22.52%) |
| Former | 11 (18.64%) | 18 (34.62%) | 29 (26.13%) |
| No | 35 (59.32%) | 22 (42.30%) | 57 (51.35%) |
| **Stage** |  |  |  |
| I |  | 34 (65.38%) |  |
| II |  | 1(1.92%) |  |
| III |  | 1(1.92%) |  |
| IV |  | 4 (7.69%) |  |
| unknown |  | 12 (23.08%) |  |
| **Pathology Subtype** |  |  |  |
| adenocarcinoma |  | 37 (71.15%) |  |
| squamous carcinoma |  | 10 (19.23%) |  |
| others |  | 5 (9.62%) |  |
| hamartoma | 4 (6.78%) |  |  |
| infection | 3 (5.08%) |  |  |
| inflammation | 29 (49.15%) |  |  |
| tuberculosis | 9 (15.25%) |  |  |
| others | 14 (23.73%) |  |  |
| **Total** | **59 (53.15%)** | **52 (46.85%)** | **111** |

**Supplement Table 3. Performance of the optimal model in the test set and the independent validation set**

| **Models** | **AUC** | **AUC**  **lower** | **AUC upper** | **Se** | **Sp** | **npv** | **ppv** | **Dataset tag** |
| --- | --- | --- | --- | --- | --- | --- | --- | --- |
| Model: *LHX9*+*GHSR*+*HOXA11*+*PTGER4-2*+*HOXB4-3* | 0.93 | 0.89 | 0.98 | 0.82 | 0.91 | 0.84 | 0.89 | Test Set |
|  | 0.82 | 0.73 | 0.90 | 0.70 | 0.82 | 0.78 | 0.70 | Independent Validation Set |

**Supplement Table 4. Performance on different cancer stages in the test set and the independent test set**

| **Models** | **Dataset tag** | **Specificity** | **Stage** | **Sensitivity** |
| --- | --- | --- | --- | --- |
| Model: *LHX9 + GHSR + HOXA1 1+ PTGER4-2 + HOXB4-3* | Test Set | 90% | 1 | 71.2% |
|  |  | 90% | 2 | 100.0% |
|  |  | 90% | 3 | 84.3% |
|  |  | 90% | 4 | 97.1% |
|  | Independent Validation Set | 80% | 1 | 68.5% |
|  |  | 80% | 2 | 100.0% |
|  |  | 80% | 3 | 100.0% |
|  |  | 80% | 4 | 72.5% |
