# Supplementary material for: Diagnosis of pulmonary nodules by DNA methylation analysis in bronchoalveolar lavage fluids
Source: Clin Epigenetics. 2021 Oct 7;13:185. doi: 10.1186/s13148-021-01163-w (PMC8499516; doi:10.1186/s13148-021-01163-w)
Supplement: Supplementary file 2 — Additional file 2. Figure S1: Methylation signals of the target CpG sites in the TCGA and the in-house databases. Figure S2: Performance of the optimal model in BALF on different pathology subtypes. Figure S3: Effect of physiological characteristics on diagnosing pulmonary nodules. [file 13148_2021_1163_MOESM2_ESM.docx]

**
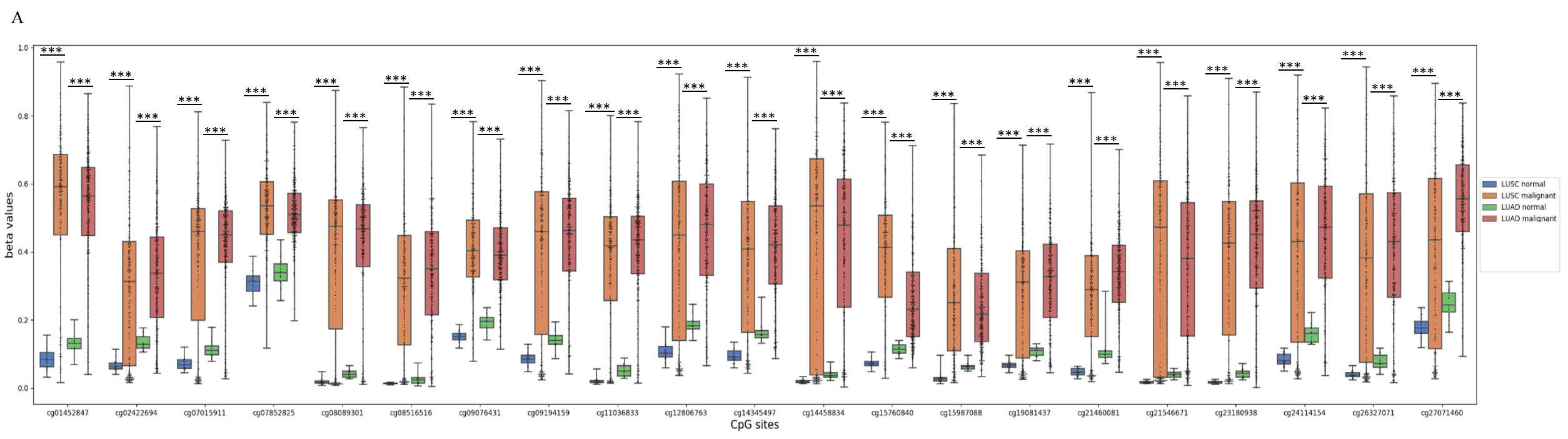
**

**
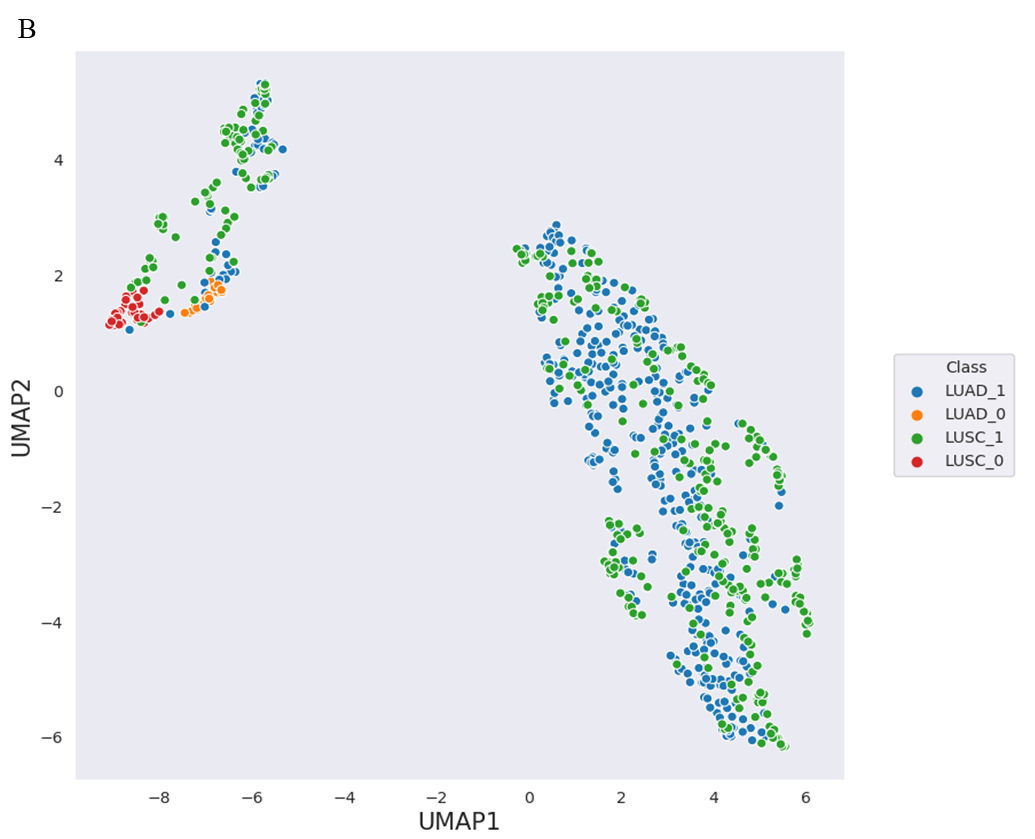
**

**
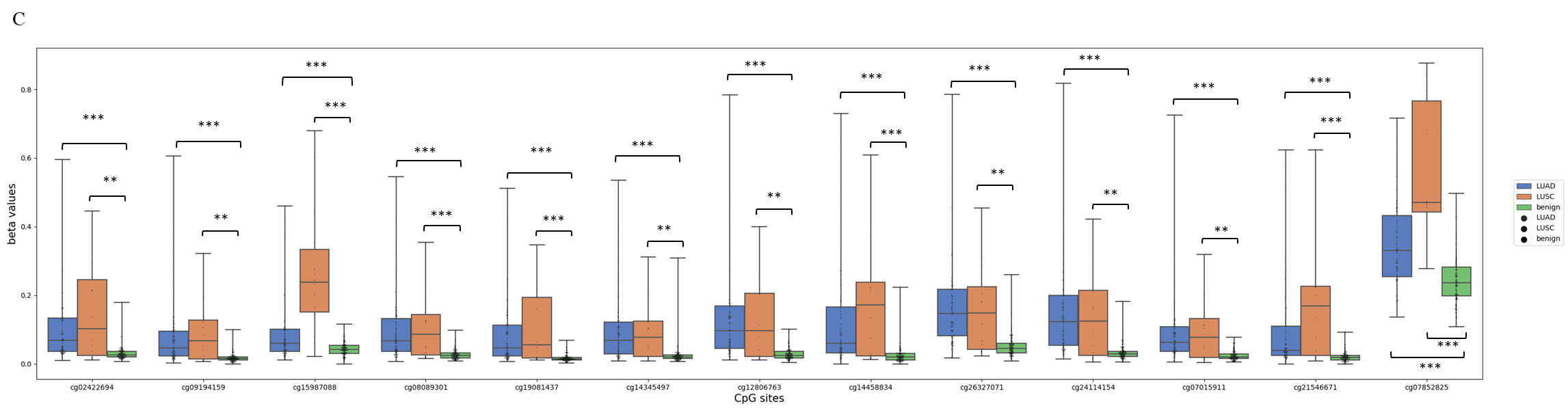
**

**
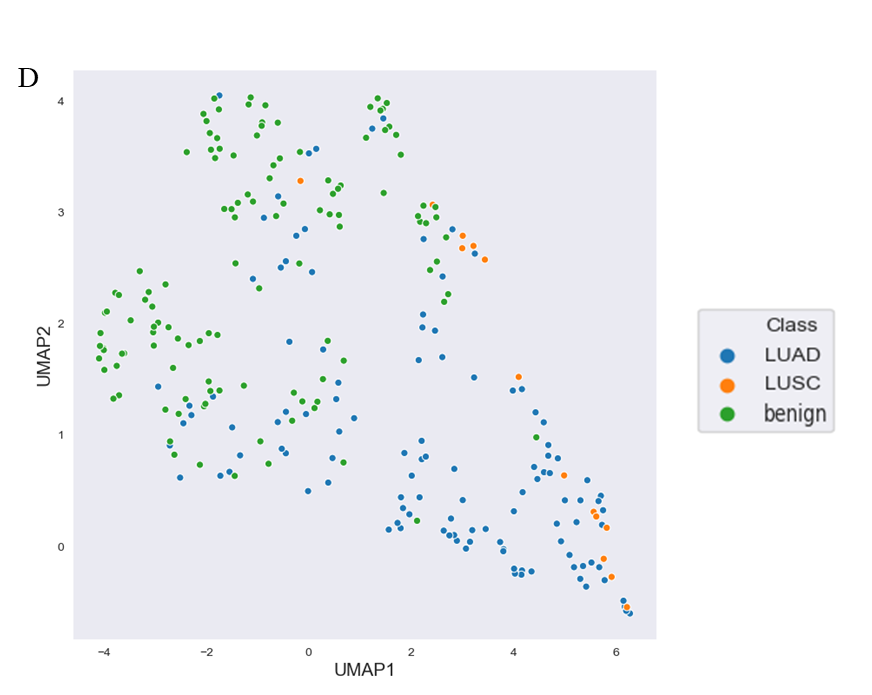
**

**
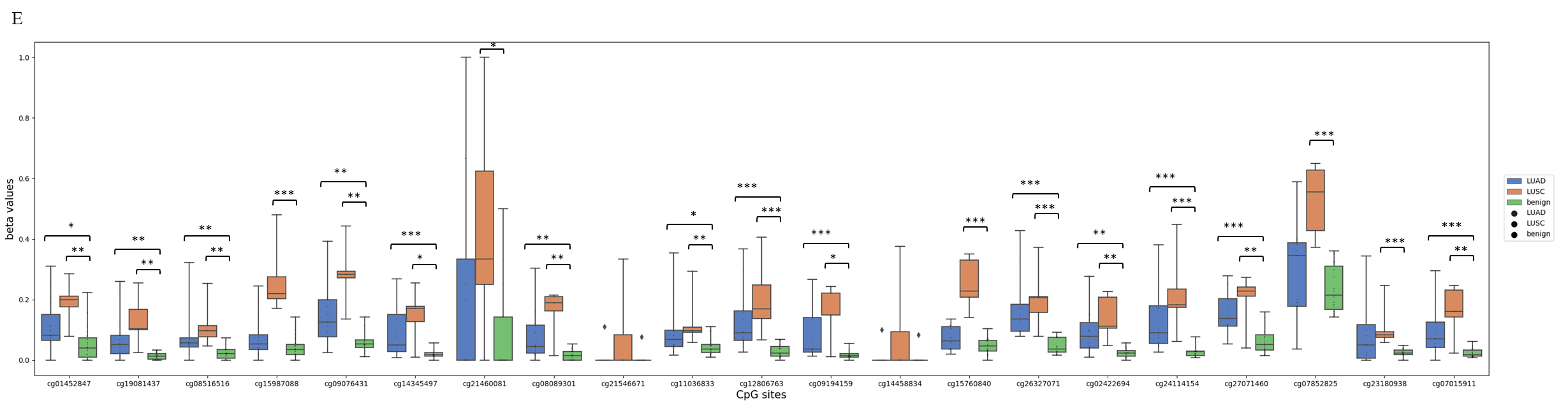
**

**
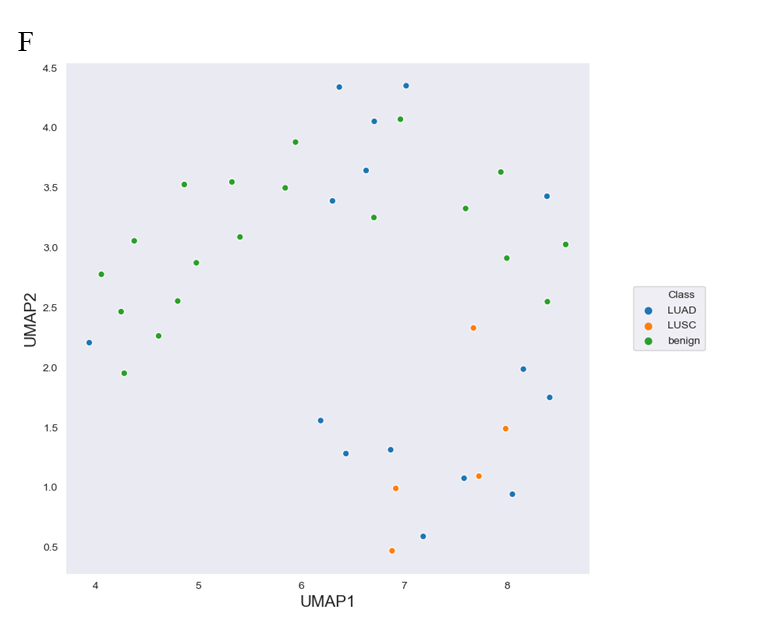
**

**Supplement Figure 1. Methylation signals of the target CpG sites in the TCGA and the in-house databases.** Twenty-one of CpG sites of the 11 target markers were found in the TCGA and the in-house database. “LUSC”, lung squamous carcinomas; “LUAD”, lung cancer adenocarcinoma; “0”，the adjacent normal tissues of lung cancer tissues; “1”，lung cancer tissues. Supplement Figure 1A and 1B, the data was from the TCGA database. Supplement Figure 1C and 1D, the data was from our previous research published in 2019 ^1^. Supplement Figure 1E and 1F, the data was from our previous research published in 2021^2^. Supplement Figure 1A, the differential methylation signals of the target CpG sites between pulmonary adjacent normal and cancer tissues in the TCGA databases. Supplement Figure 1C and 1E, the differential methylation signals of the target CpG sites between pulmonary benign and cancer tissues in the in-house databases. Y-axis, single CpG site beta value; X-axis, CpG ID. “**”, 0.001<p-value<0.01; “***”, p-value<0.001. Supplement Figure 1B, 1D and 1F, the distribution of methylation signals of the target CpG sites among different pathology subtypes in the TCGA, and the in-house databases from the published data in 2019 and 2021, respectively. (Presented by UMAP).

**
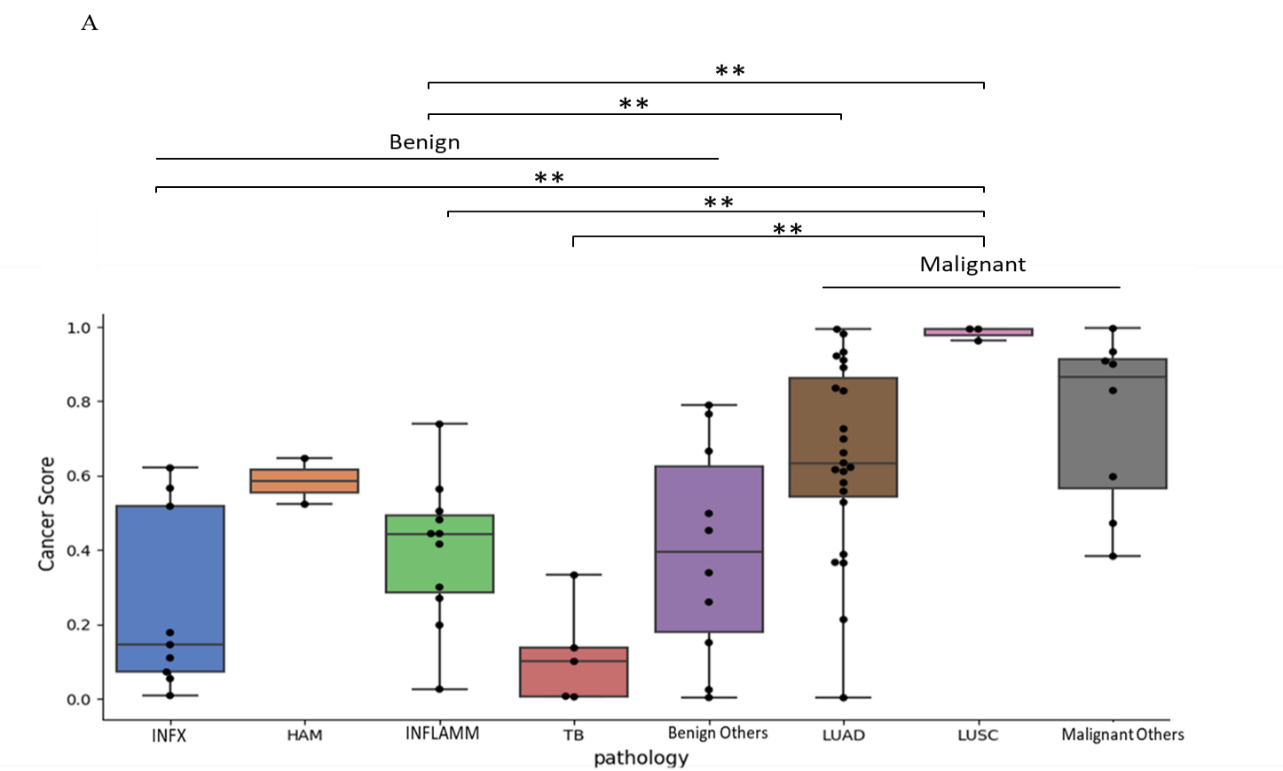
**

**
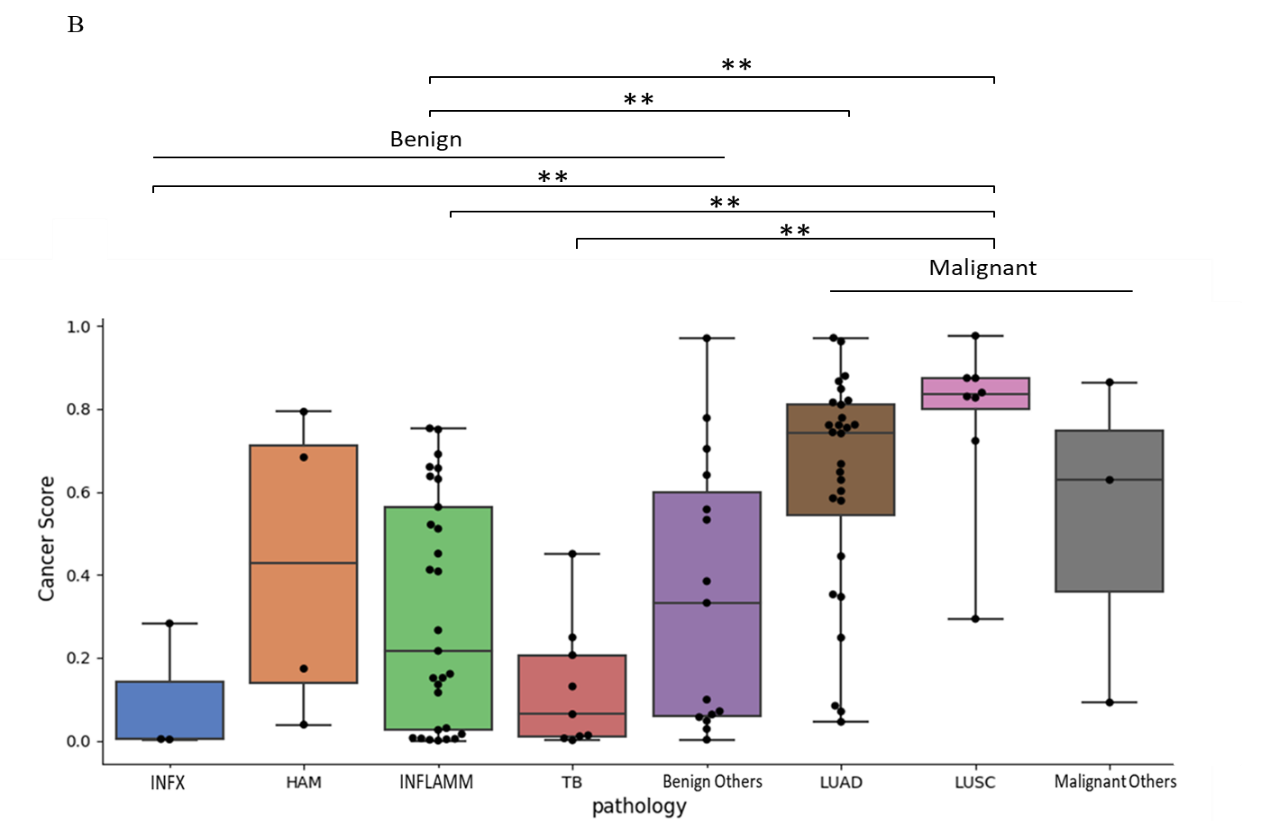
**

**Supplement Figure 2. Performance of the optimal model in BALF on different pathology subtypes**. Supplement Figure 2A and 2B present the difference of cancer score between different subtypes in the test set and the independent validation set. “LUSC”, lung squamous carcinomas; “LUAD”, lung cancer adenocarcinoma; “INFX”, pulmonary infection; “HAM”, pulmonary hamartoma; “INFLAMM”, pulmonary inflammation; “TB”, tuberculosis. Stars represent the difference between benign pulmonary diseases and either LUAD or LUSC, or between malignant nodules and different benign pathological subtypes. “**”, p-value<0.01.

**
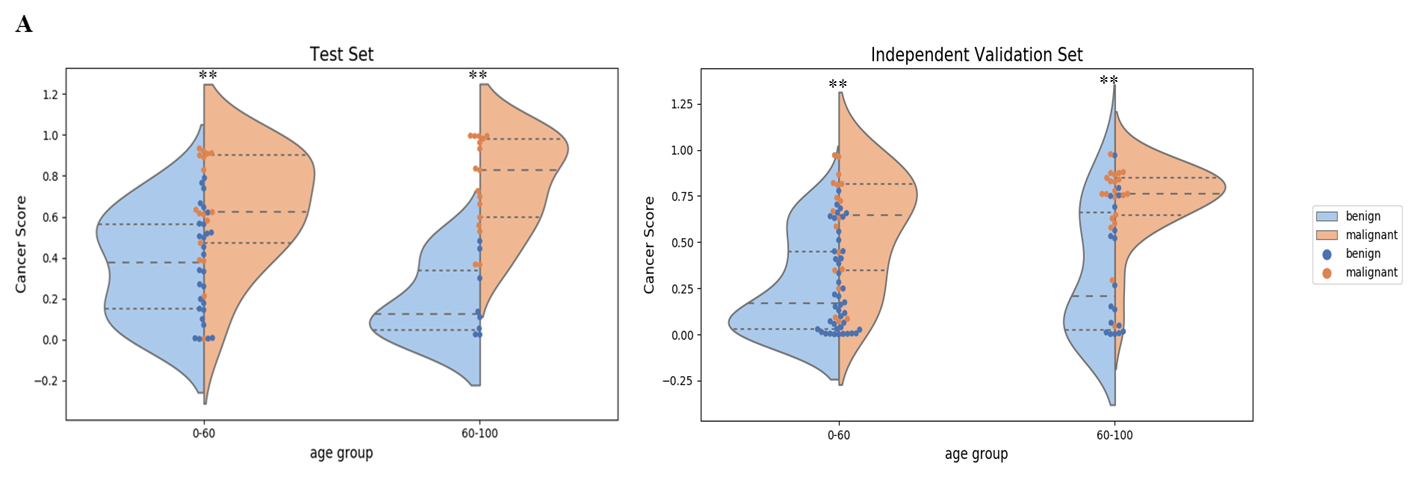
**

**
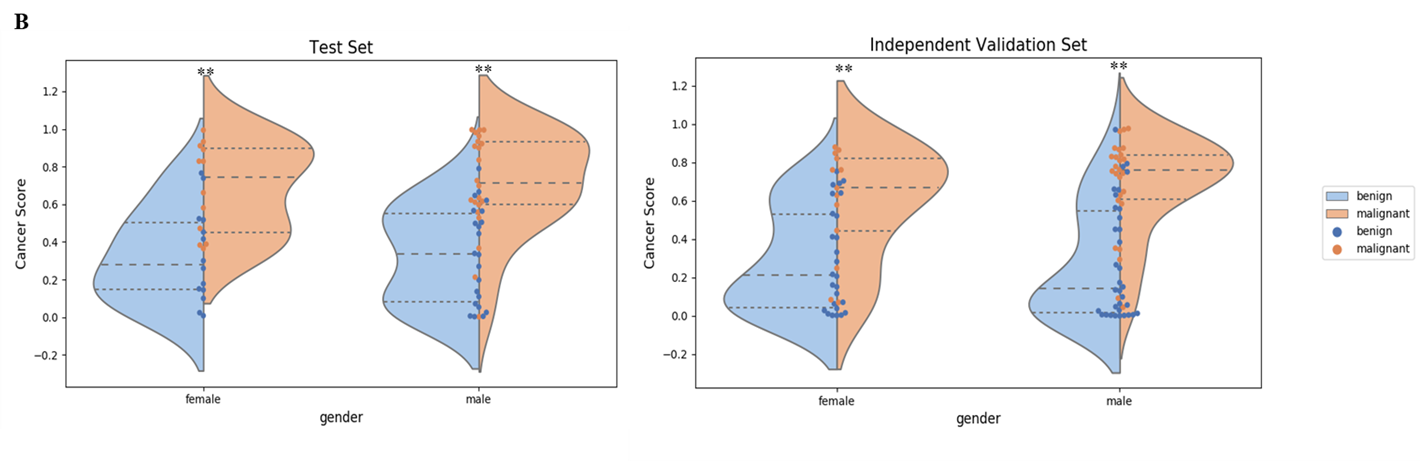
**

**
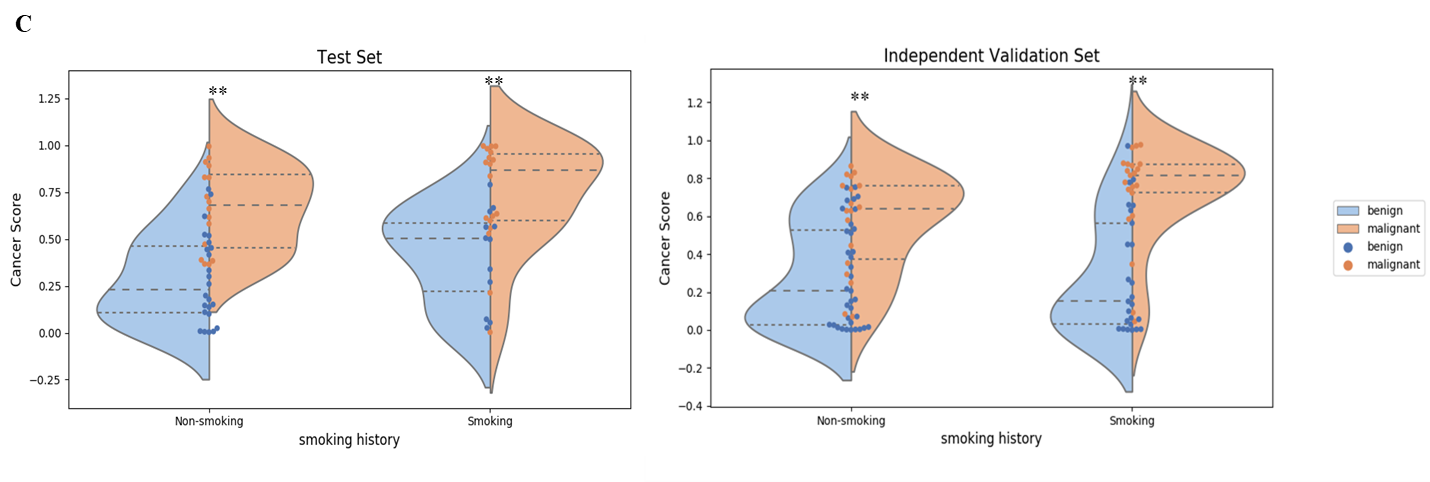
**

**
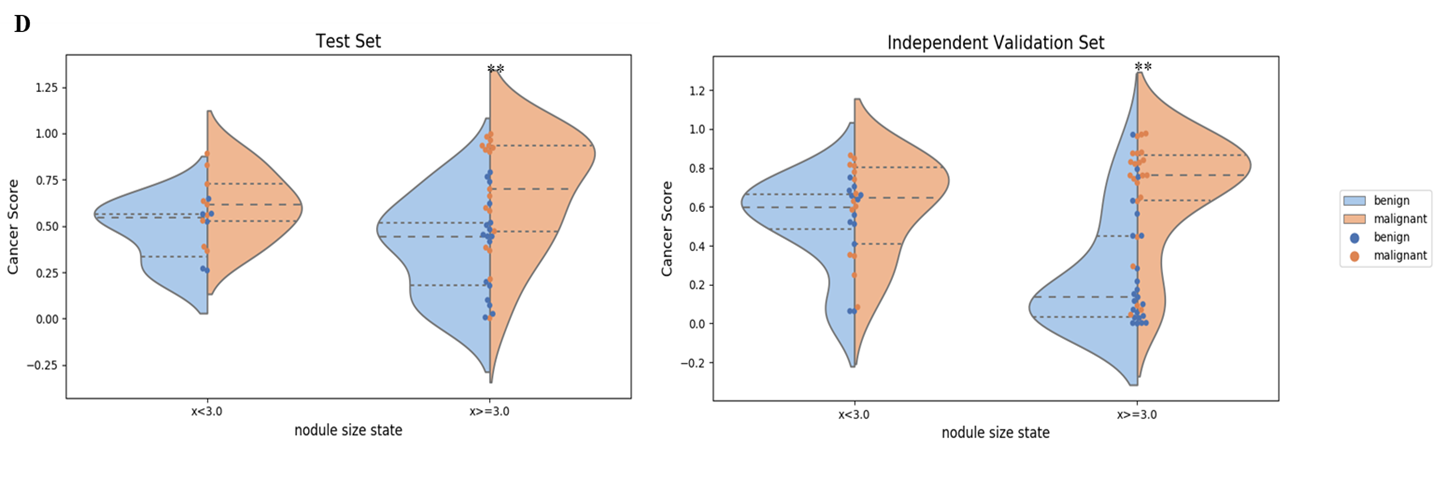
**

**
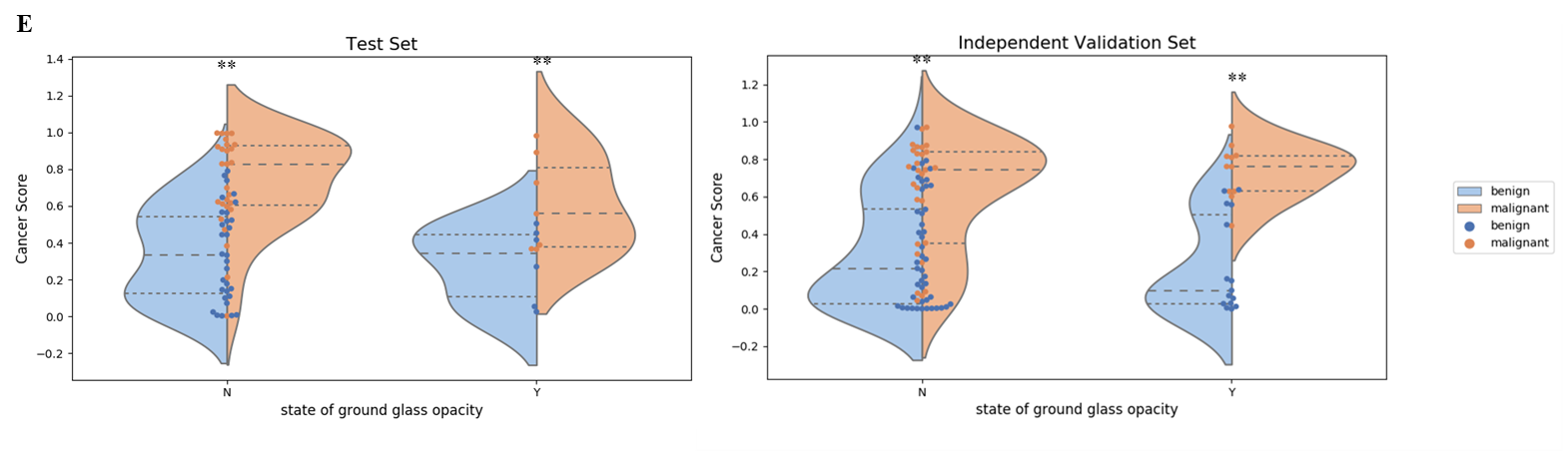
**

**Supplement Figure 3. Effect of physiological characteristics on diagnosing pulmonary nodules**

The violin plots in supplement figure 3A, 3B, 3C, 3D and 3E demonstrate the effect of age, gender, smoking history, nodule size and the solid component of the nodule on diagnosing benign and malignant pulmonary nodules, respectively. The left and right figures demonstrate the performance of the model in the test set and the independent validation set, respectively. Stars represent the difference between malignant and benign pulmonary nodules in different age groups; “*”, 0.01=<p-value<0.05; “**”, p-value<0.01;

**References:**

1. Liang W, Zhao Y, Huang W, et al: Non-invasive diagnosis of early-stage lung cancer using high-throughput targeted DNA methylation sequencing of circulating tumor DNA (ctDNA). Theranostics 9:2056-2070, 2019

2. Liang W, Chen Z, Li C, et al: Accurate diagnosis of pulmonary nodules using a non-invasive DNA methylation test. J Clin Invest, 2021
